# Cowden’s syndrome diagnosed through oral lesions: A case report

**DOI:** 10.4317/jced.58890

**Published:** 2021-11-01

**Authors:** Maureen Marshall, Doris Otero, Sven Niklander, René Martínez-Flores

**Affiliations:** 1 Unit of Oral Pathology and Oral Medicine, Faculty of Dentistry, Universidad Andres Bello, Calle Quillota 980, torre E, segundo piso, Viña del Mar, Chile; 2 Morphology Department, Faculty of Medicine, Universidad Andres Bello, Calle Quillota 980, torre D, sexto piso, Viña del Mar, Chile

## Abstract

Cowden’s syndrome (CS), also known as multiple hamartoma syndrome, is a rare autosomal dominant genodermatosis first described in 1963. It has a high penetrance in both sexes and variable phenotypes. Its origin is a PTEN (phosphatase and tensin homologue) gene mutation and affects multiple organs of endodermal, ectodermal, and mesodermal origin, resulting in the development of hamartomatous mucocutaneus lesions and an increased risk for malignancies in breast, thyroid, endometrium, kidney, colon, rectum, among other organs. The diagnosis of CS is based mainly on clinical findings and oral cavity manifestations are frequent, occurring in 80-90% of patients. This include oral and labial papillomatous papules that usually precede the development of malignant tumours. Here, we report a case of a 58-years-old male with a presumptive diagnosis of multiple “pseudofibromas” in the oral cavity that was diagnosed with CS by a dental surgeon through the identification of extra and intraoral lesions, demonstrating the importance of awareness of this entity in the dental community to improve its early diagnosis, which is vital for the early detection and treatment of malignancies.

** Key words:**Cowden’s Syndrome, Multiple Hamartoma Syndrome, PTEN Hamartoma Tumor Syndrome, Papillomatous papules.

## Introduction

Cowden’s syndrome (CS) is an autosomal dominant genodermatosis with variable expressiveness that was first recognized and described in 1963 by LLoyd and Dennis in a patient after whom the syndrome was named (1). In 1972 Weary *et al*. confirmed the existence of this new entity characterized by the formation of multiple hamartomas and neoplasms of ectodermal, endodermal and mesodermal origin, affecting different organs (2). They also reported an increased risk for the development of internal malignancies (1,3). In 1996, Nelen *et al*. located the gene responsible for CS on chromosome 10q23 and designated it as phosphatase and tensin homologue (PTEN), also termed as MMAC1 (mutated in multiple advanced cancers) or TEP1 (TGF beta-regulated and epithelial cell-enriched phosphatase) (1). Currently, CS is considered a form of the PTEN hamartoma tumor syndrome (PHTS), which also includes Bannayan-Riley-Ruvalcaba syndrome (BRRS), PTEN-related Proteus syndrome (PS), and Proteus-like syndrome (4,5).

The diagnosis of CS is based mainly on clinical findings. Even though genetic testing can be used to identify the PTEN gene mutation, in more than 20% of the patients no mutations are found (3,5). According to the International Cowden Syndrome Consortium, clinical manifestations can be grouped into pathognomonic lesions, major and minor clinical criteria (Table 1) (4,6).

**Table 1:**
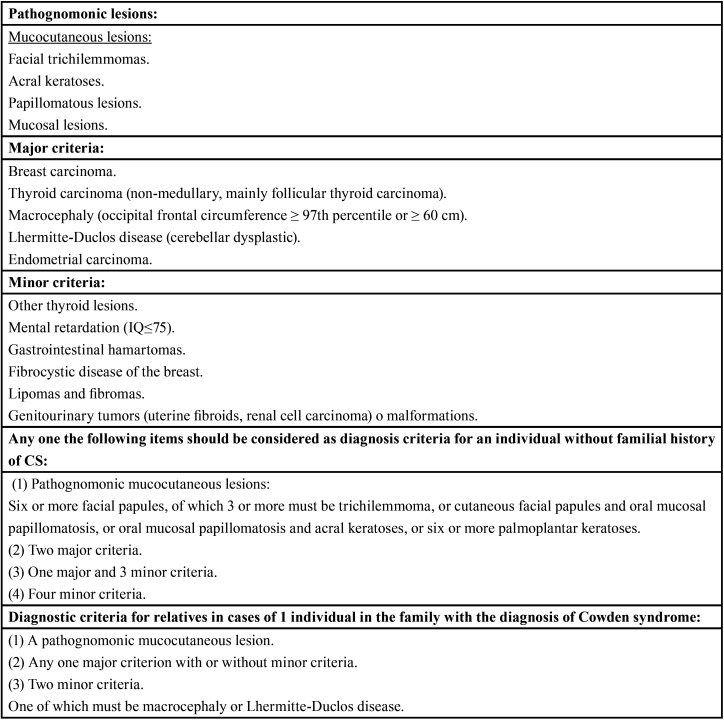
Diagnostic criteria of CS according to the International Cowden Consortium (6), revised by Pilarski and Eng (4).

Among the broad clinical spectrum, mucocutaneous lesions such as acral keratosis, trichilemmomas and oral papillomatous growths represent one of the most important group of lesions of this entity, as 99% of the patients will develop some of these during the course of the disease and they can be easily recognized by clinicians, such as dentists (7). The age of onset of these lesions ranges from birth to 46 years, with an average of 22 years (8). There is a close association of CS with the development of various malignant neoplasms, such as thyroid, breast, endometrial, colorectal and renal carcinomas, as well as melanoma (6). Lifetime risk for developing different malignant tumors ranges between 3-78%, depending on the affected organ, appearing usually years after the mucocutaneous lesions. This emphasizes the importance of theearly recognition of these lesions, which facilitates an early diagnosis of CS. This allows a close follow up and early detection of malignancies should they arise (9). Here, we report a case of CS which was diagnosed through the identification of head and neck lesions.

## Case Report

**Figure 1:**
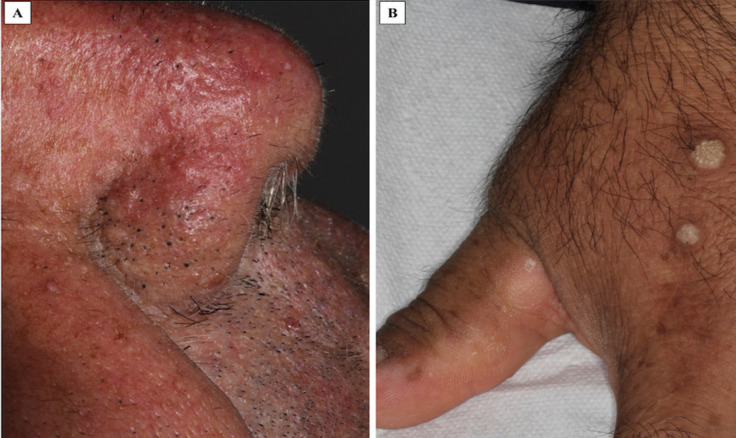
A. Multiple papillomatous lesions compatible with facial trichilemmoma around the nose. B. Warty papules found on the dorsa of the hand compatible with acral keratosis.

**Figure 2:**
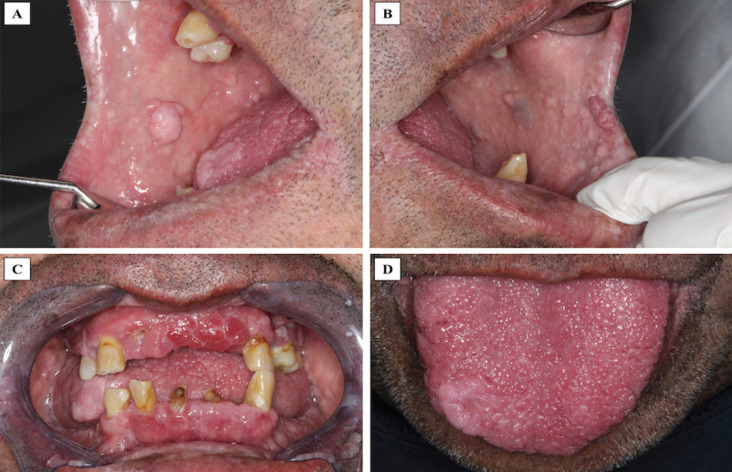
A,B. Multiple sessile pink papules with smooth surface on the right and left buccal mucosa respectively. C and D. Cobblestone appearance of multiple confluent red papules, of different size and shape on the upper, lower attached gingiva and dorsum of the tongue respectively.

**Figure 3:**
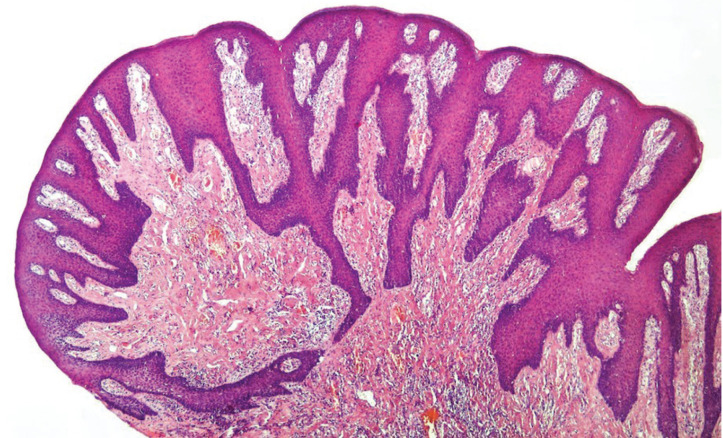
Biopsy of one of the buccal papules revealed parakeratinized and hyperplastic stratified squamous epithelium, elongated rete pegs and mild inflammation in a dense connective tissue consistent with papillomatous fibrous hyperplasia.

A 58-year-old male was referred to our oral medicine diagnostic service with a presumptive diagnosis of multiple “pseudofibromas” in the oral cavity. His medical history was not contributory. He reported the oral lesions were present since childhood and have been slowly increasing in number and size through the years. Extraoral examination revealed macrocephaly (occipital frontal circumference of 65 cm), facial trichilemmomas (Fig. 1A) and acral keratosis (Fig. 1B). Upon intraoral examination, multiple pink, sessile papules were observed on the buccal mucosa (Fig. 2A,B). In the attached gingiva and dorsum of the tongue, red papillomatous lesions with cobblestone pattern were also found (Fig. 2C,D). All these findings were suggestive of CS. A biopsy of one of the oral lesions was performed consistent with the diagnosis of papillomatous fibrous hyperplasia (Fig. 3). Thyroid ultrasound revealed uninodular goiter; and endoscopy of the upper digestive tract showed multiple hyperplasic polyps in the stomach and duodenum which were also biopsied and diagnosed as hamartomatous type polyps. With these findings, the diagnosis of CS was confirmed, and the patient was referred for genetic counselling and systemic assessment. No malignancies were detected at the time of diagnosis or when this report was submitted.

## Discussion

CS is a rare disease part of the multiple hamartoma syndrome disorder with a prevalence of 1 in 200.000 habitants (3). It is more common in Caucasians (96%); women are more affected than men (60%) and its diagnosis is commonly mistaken and delayed due to its variable expression and sometimes subtle features that can be easily missed or confused with other lesions (3,10,11) . The age of onset ranges between 4 to 75 years with a mean age of diagnosis of 39 years (10,11). According to the literature, less than 500 cases have been reported worldwide (12), many of which have been diagnosed by dental surgeons or oral pathology and medicine specialists by recognition of mucocutaneous lesions (1-3,5,7,9-13)

The present case corresponds to a white male in his 5th decade diagnosed due to an accurate extra and intraoral examination and correct identification of multiple pink colored papules of different size and shapes, facial trichilemmomas, acral keratosis and macrocephaly. Flores *et al*. and Perić *et al*. have reported similar cases of white males diagnosed with CS between 23 and 64 years (7,10). Other reports also showed that patients with CS commonly had oral mucosal papillomatosis and facial trichilemmomas at the time of diagnosis. Oral lesions can be the first sign of the disease because they develop in over 90% of the patients and are considered as a pathognomonic criteria for the diagnosis (14).

Consensus diagnostic criteria for CS were initially established in 1996, but this was prior to identification of the PTEN gene. It was not until 2013 that an evidence-based review done by *Pi*larski *et al*. led to a significant revision of the diagnostic criteria, summarized in Table 1 (4). Like other studies; the diagnosis of CS in our patient was made based on the criteria proposed by the International Cowden Syndrome Consortium (2,7,10,12,13). He fulfilled all pathognomonic lesions; six or more facial papules, three of which were trichilemmomas, orofacial papillomatosis and acral keratosis, one mayor criteria; macrocephaly, and 2 minor criteria; uninodular goiter and gastrointestinal hamartomas.

CS is associated with mutations in the tumor suppressor gene PTEN which determines loss of cell proliferation control resulting in hamartomatous growth and increased risk of breast, thyroid, colorectal, endometrial, and renal carcinomas, as well as melanoma (7,14). Stathopoulos *et al*. reported that cutaneous and mucosal lesions often manifest prior to the development of the malignant neoplasm (1). Around 67% of patients diagnosed with Cowden syndrome show thyroid pathology and up to 12 % of the patients may develop thyroid cancer throughout life; mainly follicular or papillary adenocarcinomas (1,9). Other cases also reported a history of thyroidectomy or structural thyroid alterations at the time of diagnosis, such as multinodular goiter or thyroid adenomas (2,10). Despite the late diagnosis of our patient, he only developed benign uninodular goiter, but constant thyroid ultrasounds are necessary to monitor any changes. Hence, the proper identification of these lesions, as in the present case, would allow CS to be detected in early stages, which may in turn facilitate an early diagnosis of cancer and prompt treatment, increasing survival. The gastrointestinal system in CS is also frequently affected, mainly by multiple polyps (4,8). The most common location is the colon, but other locations such stomach, duodenum, and small bowel, have also been reported (8). Our patient only agreed to have upper digestive tract endoscopy, so it is not known if he also developed colonic polyps. Risk of colorectal carcinoma is of 16% (15), so lifetime monitoring is important.

Approximately 80% of the patients with CS carry the mutation in the PTEN gene and is considered the principal form of the PHTS(4). Since the patient was an orphan, it was not possible to corroborate whether the disease was inherited in an autosomal dominant manner, or it was a mutation that occurred the novo.

Despite its recognition and description in 1963, there is a generalized lack of knowledge of CS, both in the medical and dental field. Patients can have a variety of clinical manifestations making it a diagnostic challenge. One of the most common lesions are widespread papillomatous lesions in the oral cavity, which can be easily recognized by dental surgeons or oral medicine specialists.

There is a need for awareness of this entity in the dental community. This would improve early diagnosis of CS, which is vital for the early detection and treatment of malignancies.
